# Novel breeding approach for Japanese flounder using atmosphere and room temperature plasma mutagenesis tool

**DOI:** 10.1186/s12864-019-5681-6

**Published:** 2019-04-29

**Authors:** Hou Ji-Lun, Zhang Xiao-Yan, Wang Gui-Xing, Sun Zhao-Hui, Du Wei, Zhao Ya-Xian, Si Fei, Wang Li-Yan, Xing Xin-Hui, Wang Yu-Fen

**Affiliations:** 1 Key Laboratory of Aquatic Genomics, Ministry of Agriculture, Beijing, China; 2 Beidaihe Central Experiment Station, Chinese Academy of Fishery Sciences, Qinhuangdao, China; 3 TmaxTree Biotechnology Company, Luoyang, China; 4 Key Laboratory for Industrial Biocatalysis, Ministry of Education, Department of Chemical Engineering, Tsinghua University, Beijing, China; 5 Center for Synthetic and Systems Biology, Tsinghua University, Beijing, China

**Keywords:** ARTP mutagenesis, Japanese flounder, *Paralichthys olivaceus*, Genome, Breeding

## Abstract

**Background:**

Artificial induction of mutagenesis is effective for genetic resource innovation and breeding. However, the traditional mutation methods for fish breeding are not convenient or safe for daily use. Hence, development of a simple, safe and effective mutagenesis method with a high mutation rate and applicability to multiple fish species, is needed.

**Results:**

We reported the first successful mutagenesis in a marine aquaculture fish species, Japanese flounder, *Paralichthys olivaceus*, using a novel atmosphere and room temperature plasma (ARTP) mutagenesis tool. ARTP treatment time was optimized for the fertilized eggs and sperm, respectively. Eggs fertilized for 60 min were treated by ARTP with a radio-frequency power input of 120 W, and the ARTP treatment time was 25 min. Under an ARTP radio-frequency power input of 200 W, the optimal treatment time for sperm diluted with Ringer’s solution by 1:40 *v*/v was 10 min. The ARTP-treated group presented differences in morphological traits such as body height, total length among individuals at day 90 after hatching. Whole-genome sequencing was used to reveal the mutation features of ARTP-treated individuals collected at day 120 after hatching. In total, 69.25Gb clean data were obtained from three controls and eight randomly selected ARTP-treated individuals, revealing 240,722 to 322,978 SNPs and 82,149 to 86,798 InDels located in 17,394~18,457 and 12,907~13,333 genes, respectively. The average mutation rate reached 0.064% at the genome level. Gene ontology clustering indicated that genes associated with cell components, binding function, catalytic activity, cellular process, metabolic process and biological regulation processes had higher mutation rates.

**Conclusions:**

ARTP mutagenesis is a useful method for breeding of fish species to accelerate the selection of economically important traits that would benefit the aquaculture industry, given the variety of mutations detected.

## Background

The purpose of breeding in aquaculture fish is to discover and generate economically important traits such as fast growth, disease resistance or meat quality. No matter which kind of breeding method is used, the successful breeding of improved strains basically depends on the mutations present in the base population. Mutations are the basis of genetic variation, and naturally occurring mutations play important roles in evolution. In fish, the natural mutation rate at specific loci is generally lower than 1.0 × 10^− 6^ [1]. Artificially induced mutation in fish usually employs physical radiation or chemical mutagens. One of the widely used chemical mutagens, *N*-ethyl-*N*-nitrosourea (ENU) acts as an alkylating agent, transferring its ethyl group to nucleophilic nitrogen or oxygen sites on deoxyribonucleotides, leading to base mismatching during DNA replication, and thus, it mainly induces single-base substitutions [2, 3]. The ENU has shown relatively high mutation frequency. When consider the mutation rate that based on specific gene, the mutation rate was 1 per 297 kb (0.0003%) in fugu [1], 1 per 345 kb in medaka (0.0003%) [4], 1 per 235 kb in zebrafish (0.0004%) [5] and 0.41% in grass carp (*Ctenopharyngodonidellus*) [6]. While consider mutation rate of specific loci based on phenotype, the mutation rate was 0.15% in zebrafish [7] and 0.1%~ 0.195% in medaka [8].

In addition to chemical mutagens, physical mutagens such as γ-ray, X-ray, UV, or particle radiation also play important roles in mutation induction [9]. In medaka, a specific locus test system for environmental mutagenesis was established using ^137^Cs γ-ray irradiation [10]. Moreover, atmosphere and room temperature plasma (ARTP) is a newly developed mutation system for microbes that uses a helium radio-frequency atmospheric-pressure glow discharge (RF APGD) plasma generator as its core component [11, 12]. RF APGD can be produced between two water-cooled bare-metallic electrodes driven by a radio-frequency power supply. When working, the plasma gas (helium with purity of 99.99% or better) flowing through the discharge region between the two electrodes is ionized by the externally applied RF electric field, and thus, a non-thermal plasma jet consisting of various activated chemical particles is formed at the downstream of the plasma torch nozzle exit [13, 14]. The various activated chemical particles RF APGD can alter DNA sequences by widely broken down the C-N bond between base and ribose, amino groups on the base, as well as the P-O bonds in phosphodiester [15]. Moreover, The studies of ARTP mutagenesis indicated that irradiation with ARTP is a rapid, effective, convenient, and multifaceted means of generating mutant libraries with sufficient diversity for the improvement of microbial phenotypes [14, 16, 17]. Mechanistic study on ARTP mutagenesis indicated that ARTP can cause stronger DNA damage and thereby result in higher mutation rate compared with the UV and chemical mutagens [14, 16]. ARTP has been successfully employed for mutation breeding in more than 100 kinds of microbes [17], including bacteria [18–20], fungi [21, 22], and microalgae [23–25]. However, whether ARTP can be applied to fish mutation breeding is still not clear.

Japanese flounder, *Paralichthys olivaceus*, is an economically important marine flatfish in China, and its cultural production is estimated to be 30,000 tons per year. The Japanese flounder is also a major target marine fish species for genetic and breeding studies, and many breeding methods such as gynogenesis [26, 27] and androgenesis [28] have been established. In addition, its complete genome sequence has been decoded [29]. However, decrease of Japanese flounder resource in nature, and reducing of genetic diversity in hatchery population limit progress in breeding using traditional methods [30]. Therefore, a new breeding method that could increase genetic variety is urgently needed.

In this study, we applied ARTP as a mutagenesis tool for the first time to generate mutations in Japanese flounder for breeding. The optimal ARTP mutagenesis parameters were established, and the genetic variations of ARTP-treated individuals were analyzed at the genome level using high-throughput sequencing.

## Results

### Determination of the ARTP treatment time for fertilized eggs

**Fig. 1:**
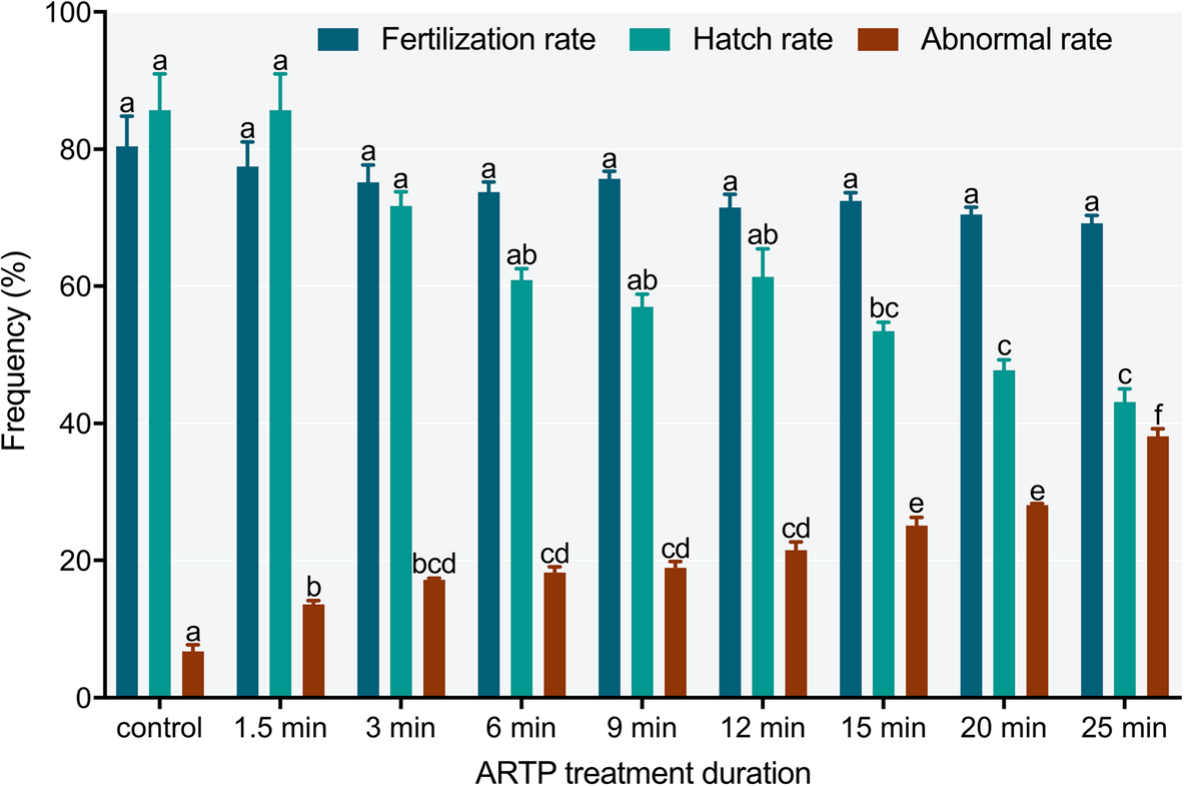
Effects of treatment duration on the fertilization, hatch and abnormal rates of Japanese flounder, *Paralichthys olivaceus*, irradiated by atmospheric and room temperature plasma (ARTP). Letters above columns indicate significant differences as determined by one-way ANOVA and LSD multiple comparisons (*P* < 0.05)

**Fig. 2:**
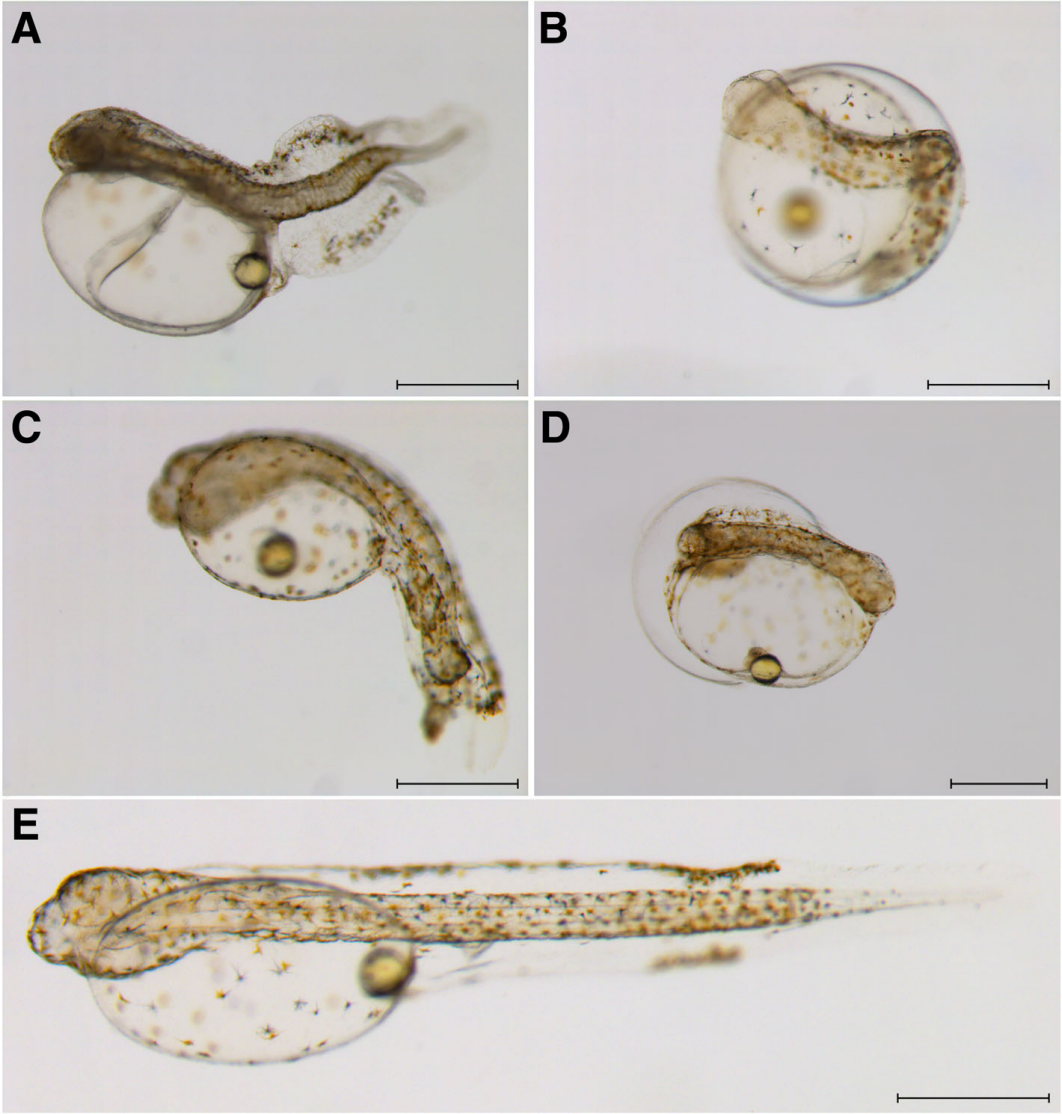
Types of abnormal larvae that hatched from ARTP-treated eggs in Japanese flounder, *Paralichthys olivaceus.* **a** Abnormal larva with short tail trunk and large cardiocoelom; (**b**) abnormal larva with camptocormia at the middle of the trunk; (**c**) abnormal larva with tail folding; (**d**) abnormal larva with shortened trunk; (**e**) normal larva from the control group. Bar indicates 500 μm

To obtain the ARTP treatment time for the fertilized eggs, 60 min after fertilization eggs were irradiated with ARTP for different treatment time periods, and the fertilization rate, hatch rate and abnormal rate were evaluated. As shown in Fig. 1, the fertilization rate ranged from 69.20 ± 2.01% to 80.43 ± 7.50%, and the difference between each group was not significant (*P* > 0.05). The hatch rates by ARTP treatment for 1.5, 3, 6, 9 and 12 min were not significantly different from each other or that of the control (*P* > 0.05). However, the hatch rates of 20 and 25 min ARTP treatment groups were significantly different from the control and the 1.5 to 12 min treatment groups (*P* < 0.05). Each treatment group included various types of abnormal larvae (Fig. 2), and the abnormal rate in each was significantly higher than that in the control group. The 25 min ARTP treatment group had an abnormal rate of 38.13 ± 1.92%, which was the highest among the treatment groups. Under an input power of 120 W, the ARTP jets increased the temperature when the treatment time period was longer than 25 min; the higher temperature was not suitable for Japanese flounder embryo development and would lead to excessive evaporation of the seawater used for egg incubation. Considering all these factors, we chose 25 min as the ARTP treatment time for Japanese flounder eggs.

### Determination of the optimal ARTP treatment time period for sperm

**Fig. 3:**
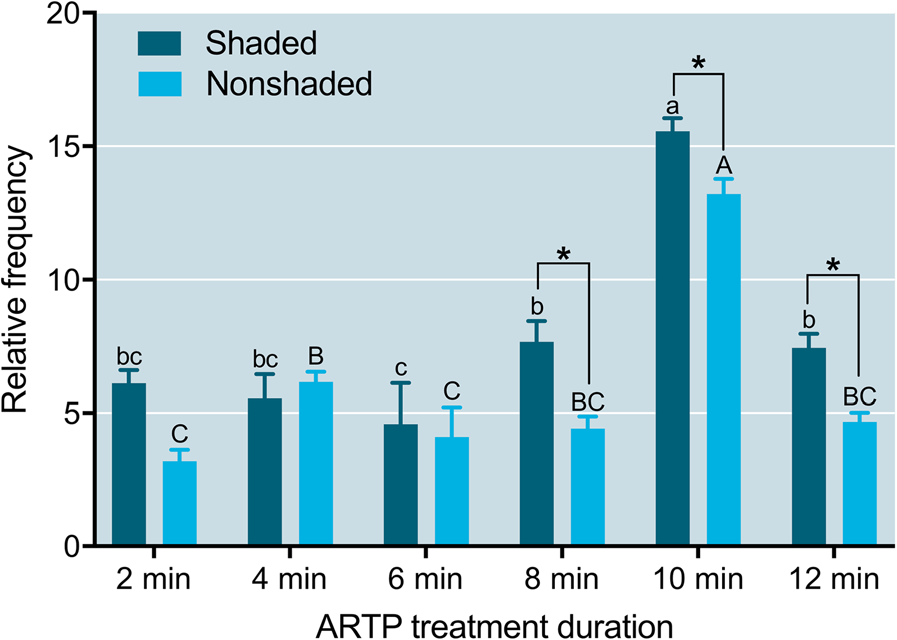
Relative abnormal rates of 1:40 diluted sperm (40X) that underwent different ARTP treatment durations and shaded or nonshaded incubation before fertilizing eggs in Japanese flounder, *Paralichthys olivaceus.* Different lowercase letters indicate significant differences among ARTP treatment durations (shaded after treatment), and different capital letters indicate significant differences among ARTP treatment durations (nonshaded after treatment), as determined by one-way ANOVA and LSD multiple comparisons (*P* < 0.05). Asterisk indicates significant difference between the shaded and nonshaded groups as determined by paired t-test (*P* < 0.05)

For 1:40 diluted sperm (40X) that were shaded after ARTP treatment, the relative abnormal rate was reduced from 2 to 6 min irradiation, then sharply increased from 8 to 10 min irradiation, and reduced again from 10 to 12 min irradiation. The 10 min ARTP treatment group had the highest relative abnormal rate, which was significantly different from those of the other groups (*P* < 0.05); the relative abnormal rates in the 8 and 12 min ARTP treatment groups were not significantly different (*P* > 0.05), but they were significantly higher than that in the 6 min group (Fig. 3). The trend of changes in the relative abnormal rate of 40X diluted sperm that were not shaded after ARTP treatment was similar to that of the shaded sperm; the only difference was that the rate ascended from 2 to 4 min ARTP irradiation and descended from 4 to 6 min irradiation (Fig. 3).

Comparing the shaded and nonshaded treatments of 40X diluted sperm, the relative abnormal rate after the shaded treatment was significantly higher than that after the nonshaded treatment (*P* < 0.05) in the 8, 10, and 12 min ARTP irradiation groups.

**Fig. 4:**
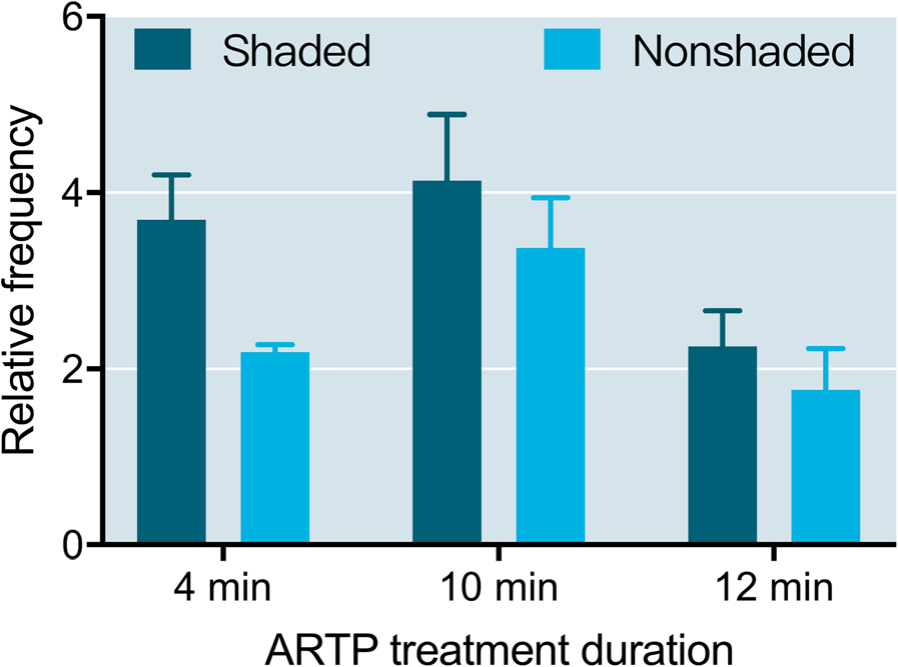
Relative abnormal rates of 1:6 diluted sperm (6X) that underwent different ARTP treatment durations and shaded or nonshaded incubation before fertilizing eggs in Japanese flounder, *Paralichthys olivaceus.* Different lowercase letters indicate significant differences among ARTP treatment durations (shaded after treatment), and different capital letters indicate significant differences among ARTP treatment durations (nonshaded after treatment), as determined by one-way ANOVA and LSD multiple comparisons (*P* < 0.05)

For 1:6 diluted sperm (6X), the differences of relative abnormal rate in groups that were shaded after ARTP treatment were not significant (*P* > 0.05). Among the nonshaded groups, the relative abnormal rate of the 10 min ARTP treatment group was significantly higher than those of the 4 and 12 min groups (*P* < 0.05). The differences between shaded and nonshaded in each ARTP treatment group were not significant (Fig. 4).

**Fig. 5:**
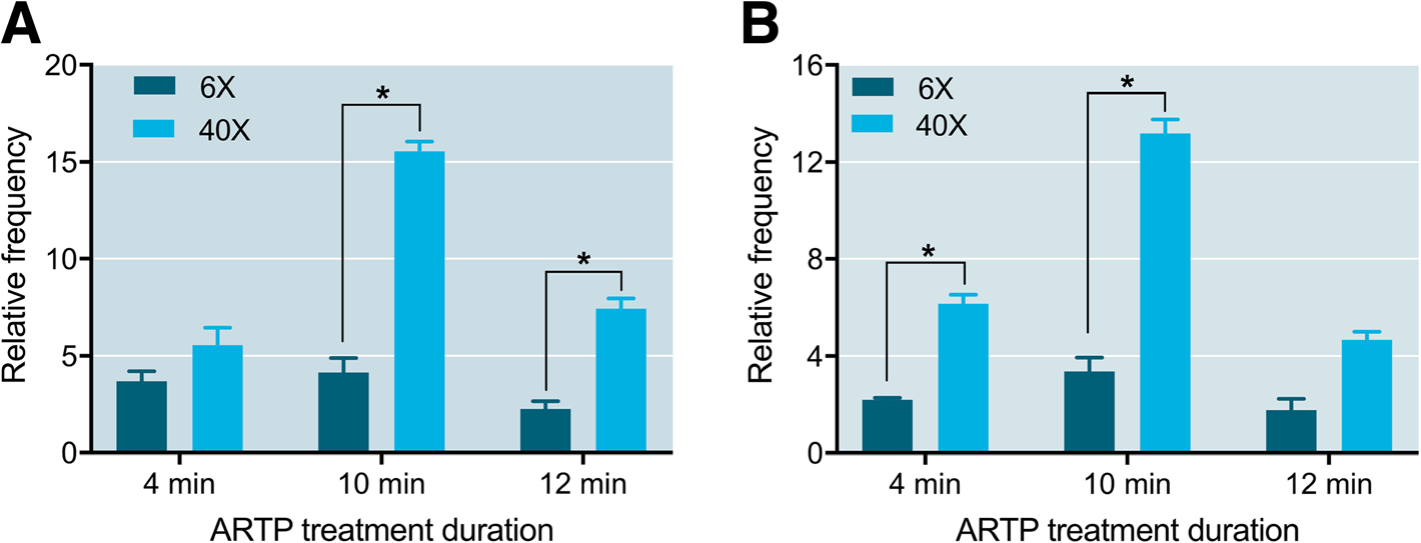
Relative abnormal rates of 1:6 diluted sperm (6X) and 1:40 diluted sperm (40X) diluted sperm that underwent shaded or nonshaded incubation after ARTP treatment in Japanese flounder, *Paralichthys olivaceus*. **a** Shaded; (**b**) nonshaded. Asterisk indicates a significant difference as determined by paired t-test (*P* < 0.05)

Comparing the 40X and 6X diluted sperm after the shaded treatment, the relative abnormal rates of the 40X diluted sperm were significantly higher than those of the 6X diluted sperm in the 10 and 12 min ARTP treatment groups (Fig. 4). In addition, after the nonshaded treatment, significant differences were found in the 4 and 10 min ARTP treatment groups, in which the 40X diluted sperm had higher relative abnormal rates than those of the 6X diluted sperm (Fig. 5).

Overall, 40X dilution and a time period of 10 min ARTP treatment are optimal for Japanese flounder sperm. Exposure of the sperm to visible light could hinder the effect of ARTP treatment to the sperms, and darkness is preferred.

### Sperm quality detection after ARTP treatment

**Fig. 6:**
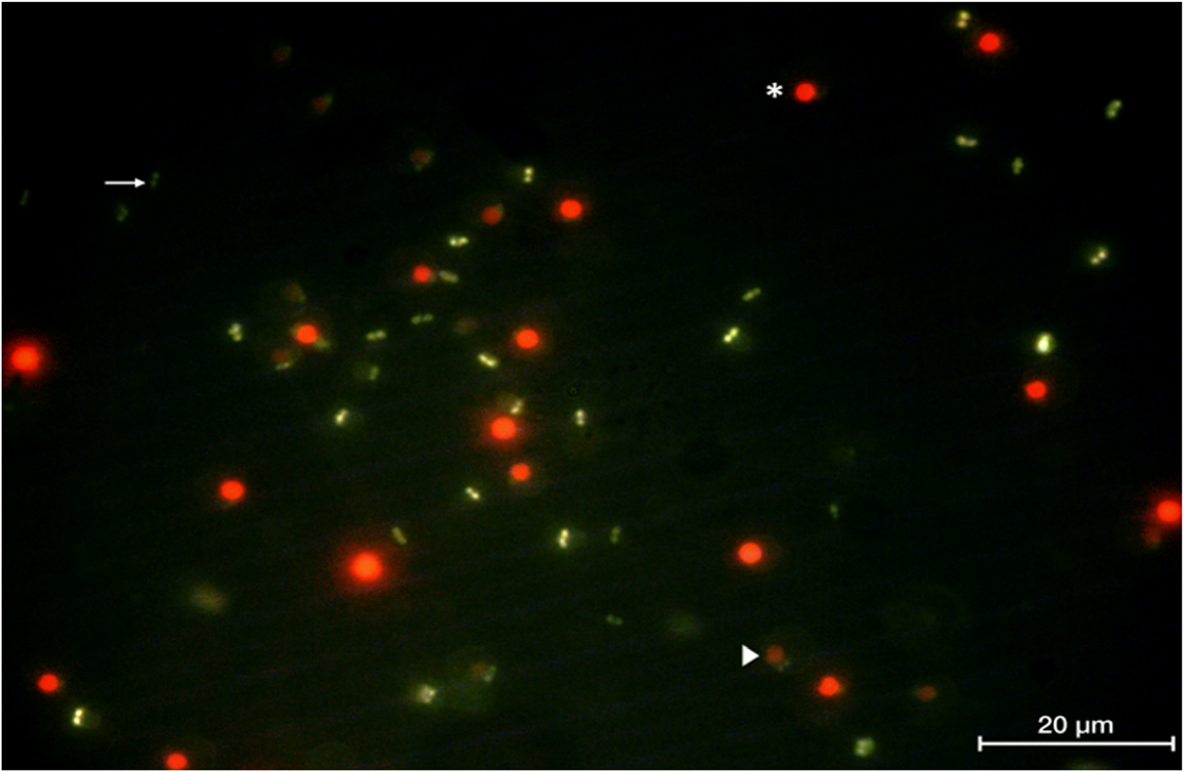
Three types of fluorescence staining patterns for sperm treated by ARTP for 4 min and double-stained by Rh123 and PI in Japanese flounder, *Paralichthys olivaceus*. Arrow indicates mitochondrially active sperm; asterisk indicates dead sperm; triangle indicates sperm with damaged plasmalemma and undamaged mitochondria. Bar indicates 20 μm

**Fig. 7:**
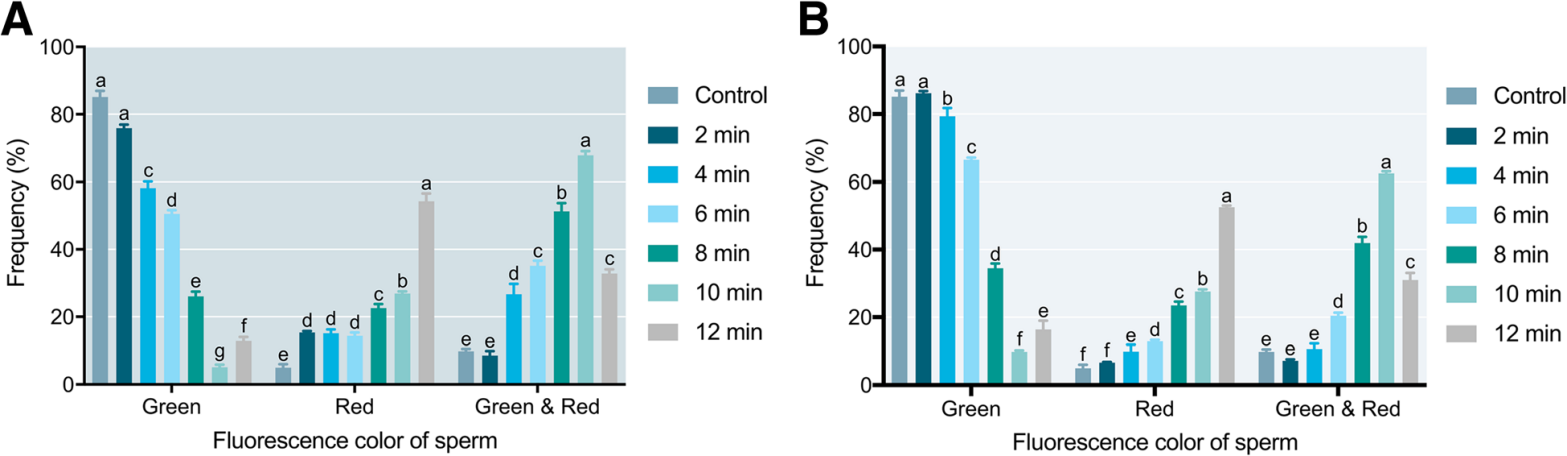
Frequency of green-, red-, and green & red-stained sperm after ARTP treatment for different durations in Japanese flounder, *Paralichthys olivaceus*. **a** Shaded; (**b**) nonshaded. Letters above columns indicate significant differences as determined by one-way ANOVA and LSD multiple comparisons (*P* < 0.05)

Three types of fluorescence staining patterns were observed for the ARTP-treated and control sperm. Sperm with active mitochondria emitted green fluorescence, dead sperm emitted red fluorescence, and sperm with damaged plasma lemma and undamaged mitochondria emitted green-red fluorescence (Fig. 6). For sperm that were shaded after the ARTP treatment, the frequency of the green fluorescence type (ratio of green fluorescence type to total sperm) in the 2 min ARTP treatment group was not different from that in the control (*P* > 0.05); while from 2 to 10 min ARTP treatment, the frequency was decreased, but it increased again in the 12 min treatment group. For the red fluorescence type, all the ARTP-treated groups had higher frequencies than that of the control (*P* < 0.05), and the 12 min treatment group showed the highest frequency of 54.31 ± 2.29%. For the green-red fluorescence type, the frequencies of the 4 to 12 min treatment groups were significantly different from that of the control (*P* < 0.05), and the highest frequency of 67.31 ± 1.27% was found in the 10 min treatment group (Fig. 7a). The frequency-change trends of different fluorescence types in nonshaded sperm were similar to those in shaded sperm (Fig. 7b).

**Fig. 8:**
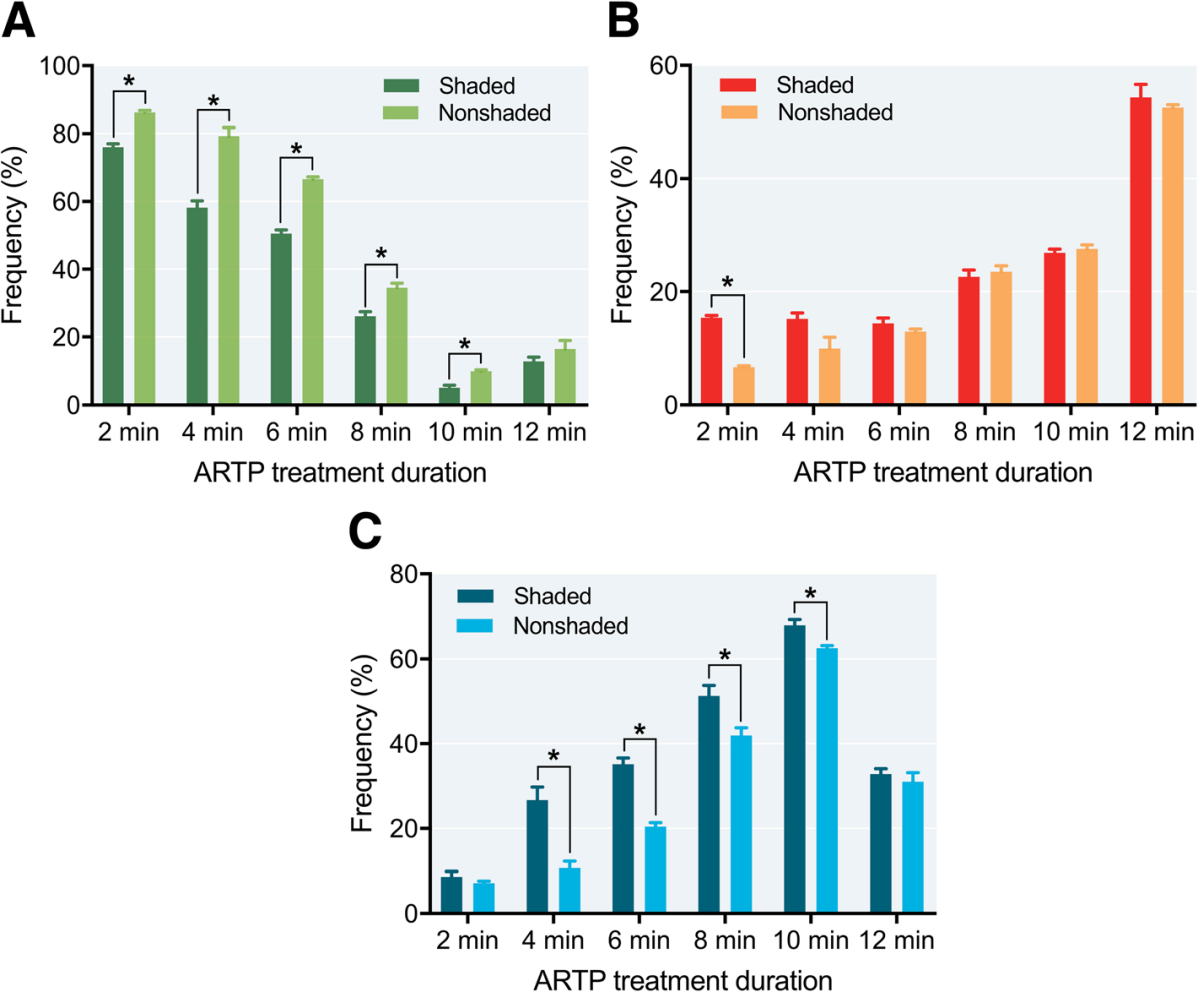
Frequency of green-, red-, and green & red-stained sperm that underwent shaded or nonshaded incubation after ARTP treatment in Japanese flounder, *Paralichthys olivaceus*. **a** Green-stained sperm; (**b**) red-stained sperm; (**c**) green & red-stained sperm. Asterisk indicates a significant difference as determined by paired t-test (*P* < 0.05)

Comparing the shaded and nonshaded treatments, except in the 12 min group, the groups (ARTP treatment for 2 to 10 min) were significantly different (*P* < 0.05) in terms of frequency of green fluorescence type (Fig. 8a). Only the 2 min ARTP treatment group was significantly different (*P* < 0.05) in frequency of red fluorescence type (Fig. 8b). The 4 to 10 min ARTP groups were significantly different (*P* < 0.05) in frequency of green-red fluorescence type (Fig. 8c).

### Morphological characteristics

**Fig. 9:**
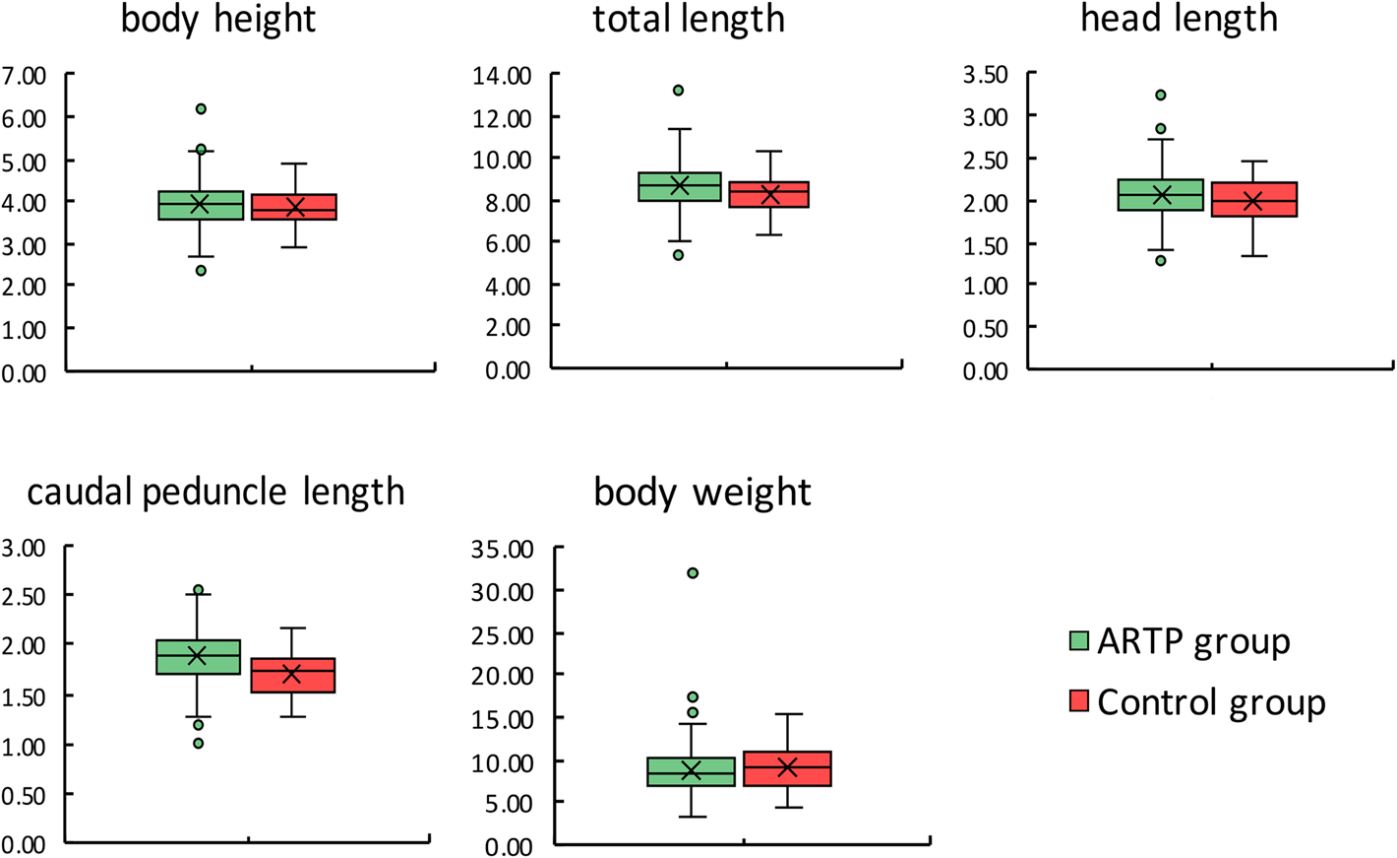
Box-plots of body height, total length, head length, caudal peduncle length and body weight in ARTP and control group in Japanese flounder, *Paralichthys olivaceus*. Colored dots indicate outliers with values greater than 1.5 times the interquartile range

Each 180 individuals from ARTP treatment and control groups were measured for body weight, total length, head length, body height and caudal peduncle length at day 90 after hatch. Among all the traits measured, the total length, head length and caudal peduncle length were significantly different (*P* < 0.05) between the ARTP-treated group and control group. The differences in body weight and body height between the groups were not significant (*P* > 0.05) (Table 1). While the maximum value of each trait in the ARTP group was higher than that in the control group, and the minimum values in the ARTP group were lower than those in the control group. Except for the caudal peduncle length trait, the ARTP-treated group had higher CVs, and many morphological differences between individuals were present within the ARTP-treated group (Table 1) (Fig. 9).

**Table 1:** Morphological traits of ARTP treated and control Japanese flounder, *Paralichthys olivaceus*

	Mean ± SD		CV (%)		Max		Min	
	ARTP group	Control group	ARTP group	Control group	ARTP group	Control group	ARTP group	Control group
Body weight (g)	8.82 ± 3.22^a^	9.02 ± 2.64^a^	36.53	29.08	32.10	15.30	3.20	4.40
Total length (cm)	8.65 ± 1.10^a^	8.28 ± 0.96^b^	12.74	11.63	13.23	10.35	5.29	6.27
Head length (cm)	2.08 ± 0.29^a^	1.98 ± 0.26^b^	13.83	13.04	3.23	2.48	1.30	1.34
Body height (cm)	3.92 ± 0.56^a^	3.84 ± 0.45^a^	14.32	11.77	6.21	4.90	2.32	2.94
Caudal peduncle length (cm)	1.89 ± 0.25^a^	1.71 ± 0.24^b^	13.09	14.11	2.55	2.16	1.00	1.27

Note: different superscript letters between columns indicates significant differences as determined by paired t-test (*P* < 0.05)

### Genome-wide analysis of ARTP mutant individuals

In total, we obtained 69.25Gb clean data from three controls and eight randomly selected ARTP-treated individuals, and the GC contents ranged from 41.42 to 42.10%. All samples were of high quality (Q20 ≥ 94.90%, Q30 ≥ 87.09%) (Table 2). The average mapping rate of sample to the reference genome sequence was 96.22%, and the average depth was 9.77X (Table 3).

**Table 2:** Genome sequencing data and quality of each sample

Sample	Raw Base (bp)	Clean Base(bp)	Effective Rate(%)	Error Rate(%)	Q20(%)	Q30(%)	GC Content(%)
c1	7,062,951,300	7,049,850,900	99.81	0.04	96.20	90.28	41.89
c2	5,863,885,800	5,846,086,200	99.70	0.04	96.45	90.91	41.99
c3	5,782,930,200	5,770,443,900	99.78	0.04	96.59	91.13	41.95
m1	6,310,089,300	6,294,106,500	99.75	0.04	96.46	90.88	41.99
m2	6,472,890,300	6,459,912,300	99.80	0.04	96.59	91.12	41.64
m3	6,625,437,000	6,594,946,500	99.54	0.04	96.21	90.34	42.05
m4	6,345,791,100	6,330,096,600	99.75	0.04	96.30	90.50	41.97
m5	6,039,080,700	6,017,527,500	99.64	0.05	94.90	87.09	42.02
m6	6,755,125,800	6,715,168,200	99.41	0.04	96.50	94.72	42.10
m7	6,306,369,000	6,287,940,000	99.71	0.04	96.52	90.95	41.94
m8	5,902,783,200	5,886,753,900	99.73	0.04	97.32	93.14	41.42

**Table 3:** Summary of assembly results

Sample	Total reads	Mapped reads	Mapping rate (%)	Average depth (X)	Coverage at least 1X (%)	Coverage at least 4X (%)
c1	46,999,006	45,302,172	96.39	10.54	99.33	95.68
c2	38,973,908	37,467,504	96.13	9.15	99.19	91.90
c3	38,469,626	37,028,773	96.25	9.11	99.18	91.75
m1	41,960,710	40,458,673	96.42	9.68	99.25	93.58
m2	43,066,082	41,487,247	96.33	9.89	99.29	94.35
m3	43,966,310	42,412,162	96.47	10.12	99.30	94.68
m4	42,200,644	40,611,165	96.23	9.76	99.27	93.83
m5	40,116,850	38,536,371	96.06	9.43	99.21	92.64
m6	44,767,788	42,971,134	95.99	10.86	99.36	96.06
m7	41,919,600	40,377,059	96.32	9.69	99.25	93.50
m8	39,245,026	37,616,558	95.85	9.20	99.21	92.34

Compared with the reference genome sequence, 3,089,328, 2,828,464 and 2,822,287 SNPs were detected in controls 1, 2 and 3. For the ARTP-treated individual m1 to m8, the detected SNP number ranged from 2,873,580 to 3,068,638 (Table 4). In the case of InDels, 482,567, 433,478, and 433,286 were detected in controls 1, 2 and 3, respectively, and 434,536 to 488,874 were detected in the ARTP-treated individuals m1 to m8 (Table 5).

**Table 4:** SNP information for control and ARTP treated samples after calling

Sample	Upstream	Exonic	Intronic	Splicing	Downstream	upstream/downstream	Intergenic	Total	ts	tv	ts/tv
c1	116,469	116,181	1,356,072	272	101,018	5849	1,393,467	3,089,328	1,676,361	1,412,967	1.186
c2	106,347	109,337	1,241,373	250	92,833	5258	1,273,066	2,828,464	1,537,682	1,290,782	1.191
c3	105,941	108,654	1,239,371	262	92,870	5236	1,269,953	2,822,287	1,534,177	1,288,110	1.191
m1	109,996	112,412	1,288,306	263	95,991	5487	1,321,804	2,934,259	1,595,108	1,339,151	1.191
m2	112,947	111,782	1,314,387	259	98,438	5714	1,351,103	2,994,630	1,623,581	1,371,049	1.184
m3	113,352	114,531	1,317,274	272	98,624	5595	1,352,071	3,001,719	1,630,355	1,371,364	1.188
m4	111,372	112,731	1,296,977	270	96,508	5583	1,326,500	2,949,941	1,601,682	1,348,259	1.187
m5	107,632	109,741	1,263,174	254	94,376	5404	1,292,999	2,873,580	1,560,292	1,313,288	1.188
m6	115,586	116,261	1,347,778	276	100,946	5744	1,382,047	3,068,638	1,666,239	1,402,399	1.188
m7	109,894	111,811	1,286,623	269	96,192	5416	1,320,482	2,930,687	1,592,922	1,337,765	1.19
m8	108,839	105,835	1,265,898	243	95,682	5439	1,301,310	2,883,246	1,562,074	1,321,172	1.182

**Table 5:** InDel information for control and ARTP treated samples after calling

Sample	Upstream	Exonic	Intronic	Splicing	Downstream	Upstream/Downstream	Intergenic	Total
c1	19,889	3412	220,932	182	18,222	1003	218,927	482,567
c2	17,988	3139	198,478	162	16,294	884	196,533	433,478
c3	17,785	3140	198,681	159	16,398	898	196,225	433,286
m1	18,655	3251	207,611	179	17,114	944	205,527	453,281
m2	19,241	3251	212,754	165	17,651	979	211,069	465,110
m3	19,398	3406	214,410	181	17,663	971	211,730	467,759
m4	18,900	3281	208,851	170	17,254	916	206,160	455,532
m5	17,825	3188	198,936	162	16,571	892	196,962	434,536
m6	20,213	3487	224,314	186	18,366	1006	221,302	488,874
m7	18,525	3237	207,038	174	17,040	929	204,741	451,684
m8	18,423	2985	203,099	156	16,988	941	201,253	443,845

**Fig. 10:**
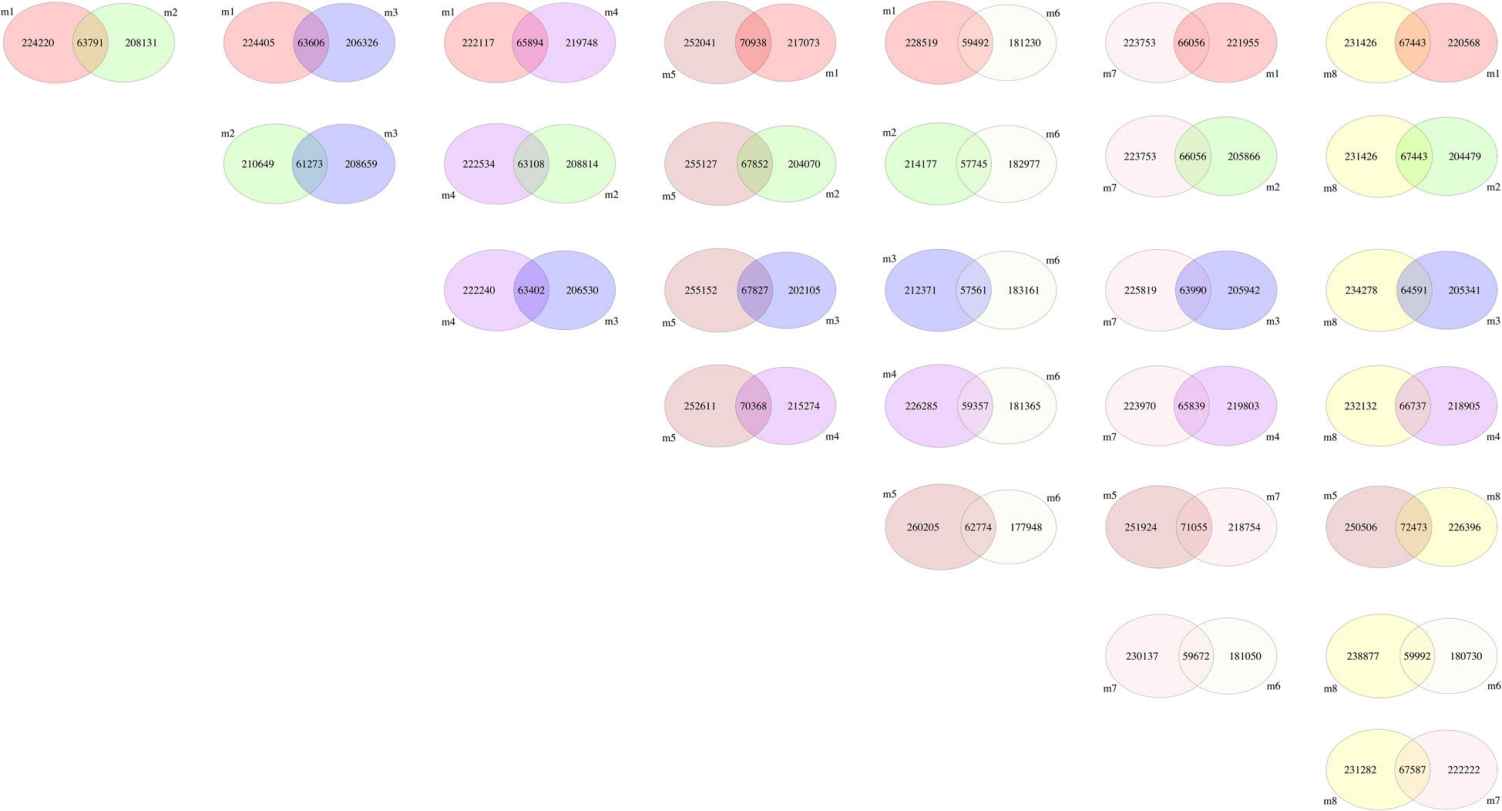
SNP comparison among individuals of ARTP-treated Japanese flounder, *Paralichthys olivaceus.* The difference of SNP for each two individuals was represented using single Venn diagram. For example, individual m1 compared with m2, 224,220 was the number of unique SNP of m1 when compared with m2, 208,131 was the number of unique SNP of m2 when compared with m1, and 63,791 was the SNP number that shared by m1 and m2

**Fig. 11:**
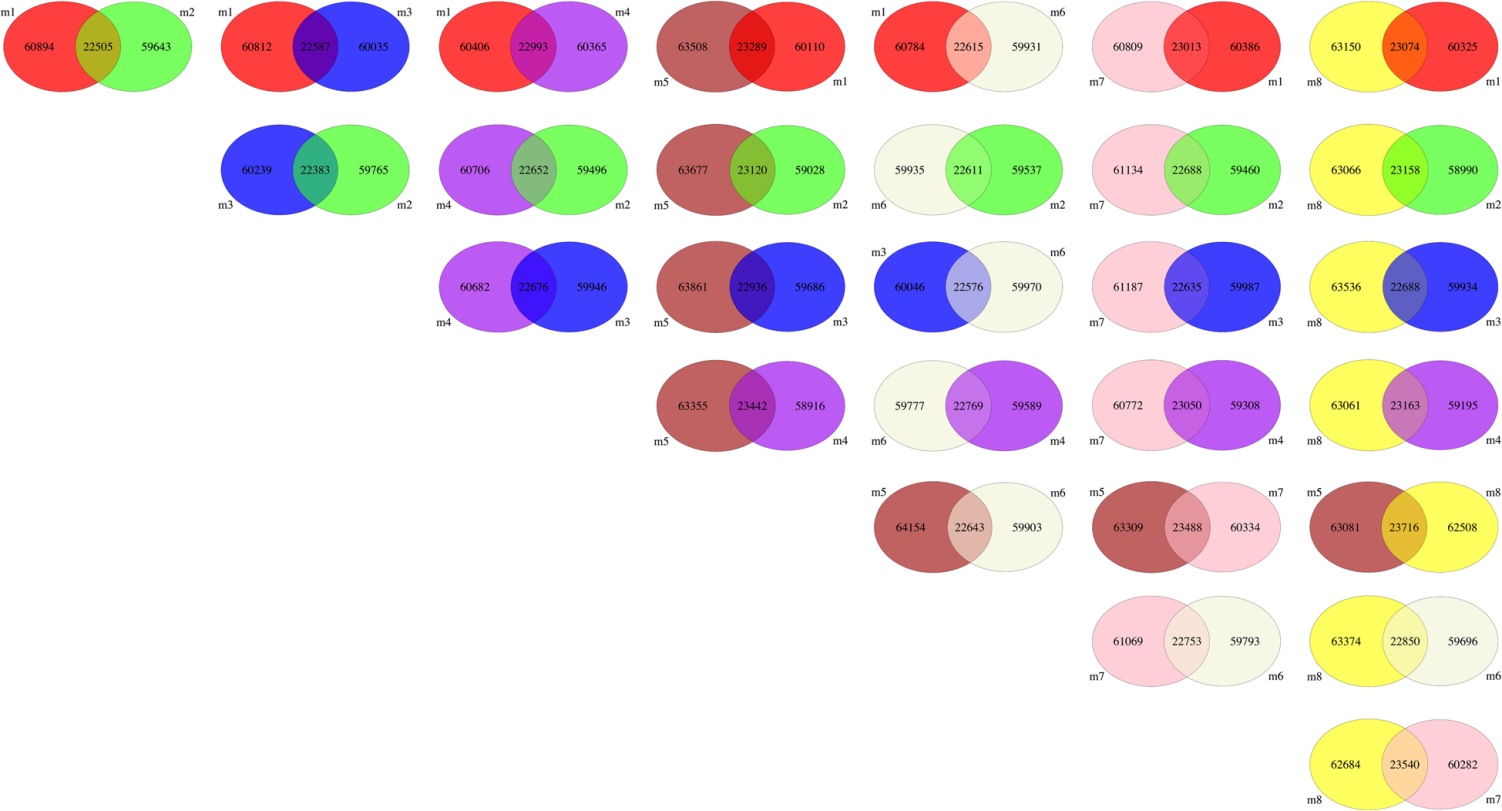
InDel comparison among individuals of ARTP-treated Japanese flounder, *Paralichthys olivaceus.* The difference of InDel for each two individuals was represented using single Venn diagram. For example, individual m1 compared with m2, 60,894 was the number of unique InDel of m1 when compared with m2, 59,643 was the number of unique InDel of m2 when compared with m1, and 22,505 was the InDel number that shared by m1 and m2

Next, ARTP-treated individuals m1-m8 were compared with controls 1, 2 and 3 to remove the shared SNPs and InDels. The results of this comparison showed that the SNP number of the ARTP-treated samples ranged from 240,722 to 322,978, and the InDel number ranged from 82,149 to 86,798 (Table 6). Comparing individual samples, the number of SNPs shared by sample pairs ranged from 57,561 (m3 vs m6) to 72,473 (m5 vs m8) (Fig. 10), and the shared InDel number ranged from 22,383 (m3 vs m2) to 23,716 (Fig. 11)

**Table 6:** SNP and InDel number of ARTP treated samples after removing shared types with controls

Sample	No. of SNP	No. of InDel
m1	288,011	83,400
m2	271,922	82,149
m3	269,932	82,623
m4	285,635	83,359
m5	322,978	86,798
m6	240,722	82,545
m7	289,809	83,822
m8	298,869	86,224

**Fig. 12:**
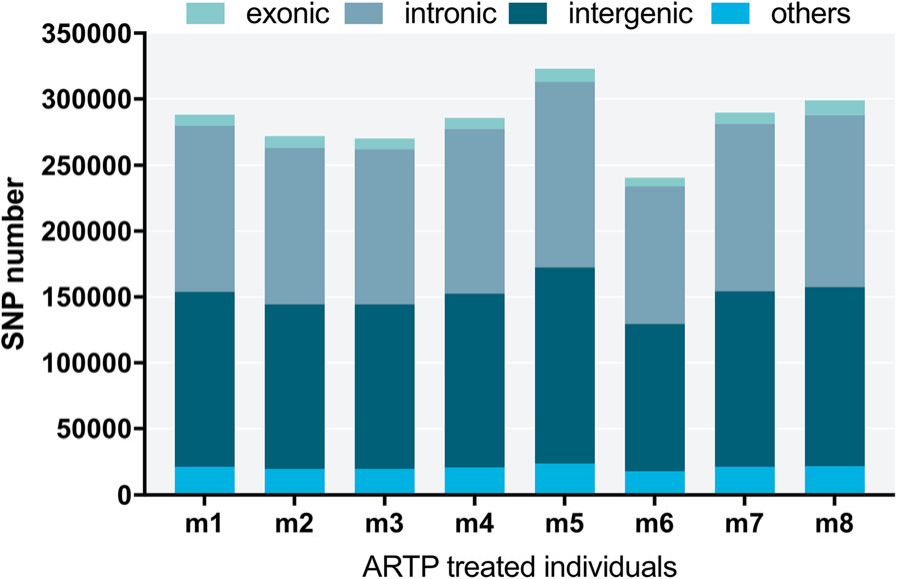
Numbers of different types of SNP in individual ARTP-treated Japanese flounder, *Paralichthys olivaceus*

**Fig. 13:**
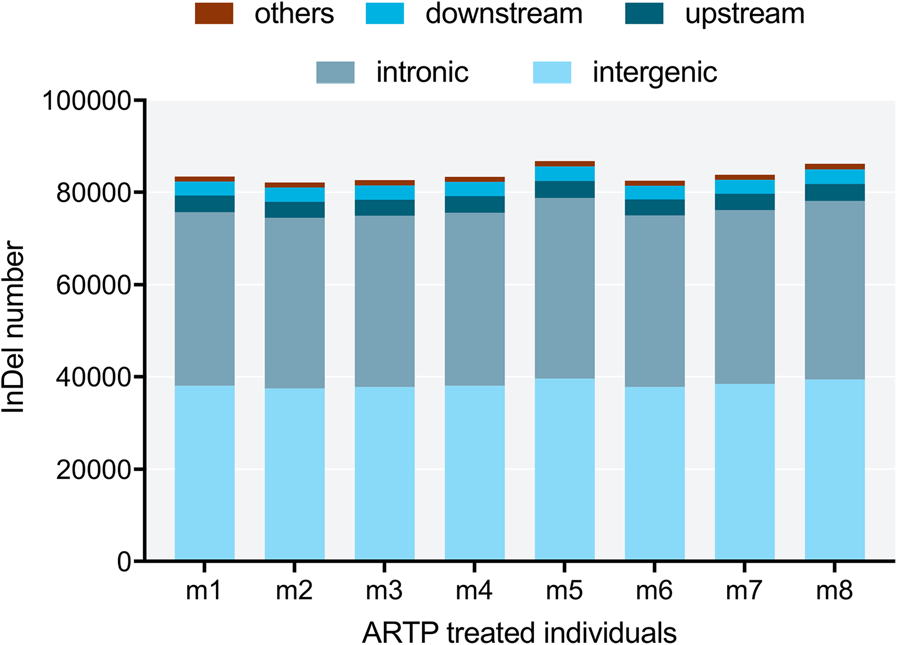
Numbers of different types of InDels in individual ARTP-treated Japanese flounder, *Paralichthys olivaceus*

In the ARTP-treated samples, a mean percentage of 45.96% of SNPs were located in intergenes, while 43.60 and 3.07% were in introns and exons. SNP types such as upstream, downstream, upstream/downstream and splicing that we classified as ‘others’ accounted for7.37% (Fig. 12). The majority of InDel types in the ARTP-treated samples were intergenic and intronic, which had mean percentages of 45.70 and 44.99%, respectively, followed by upstream (4.28%) and downstream (3.66%) types. Other 1.37% InDel types included upstream/downstream, frameshift deletion, frameshift insertion, frameshift substitution, nonframe shift deletion, nonframe shift insertion, nonframe shift substitution, splicing, stop gain SNV, and stop loss SNV (Fig. 13).

### Functional clustering of the mutant genes

**Fig. 14:**
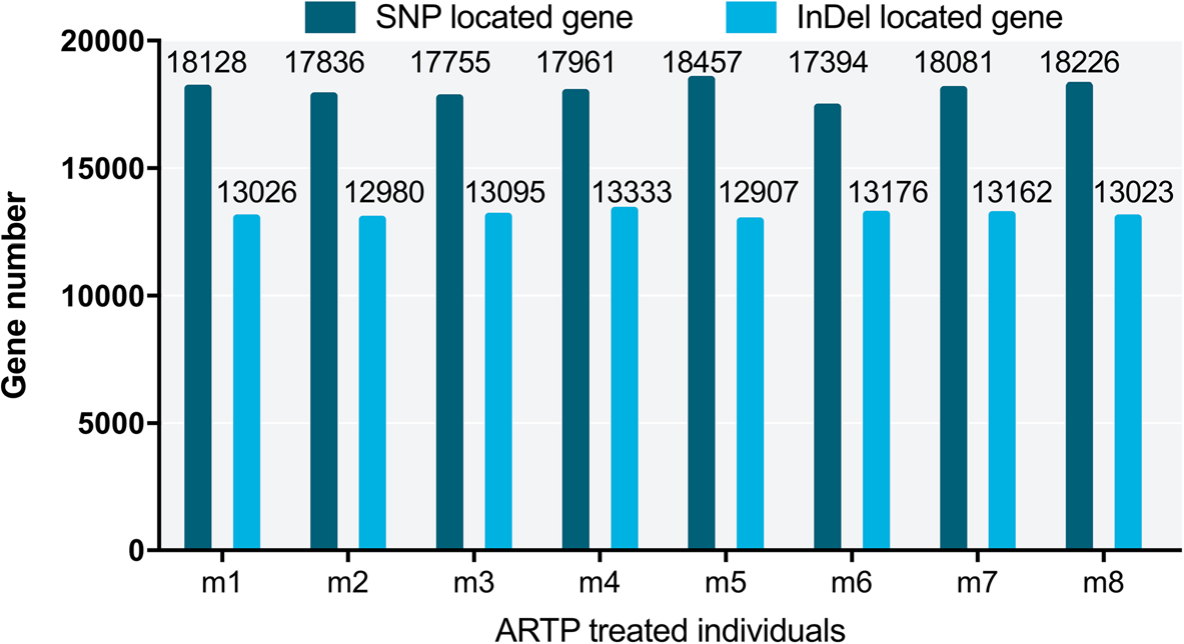
SNP and Indel distribution in the genomes of individual Japanese flounder, *Paralichthys olivaceus*

**Fig. 15:**
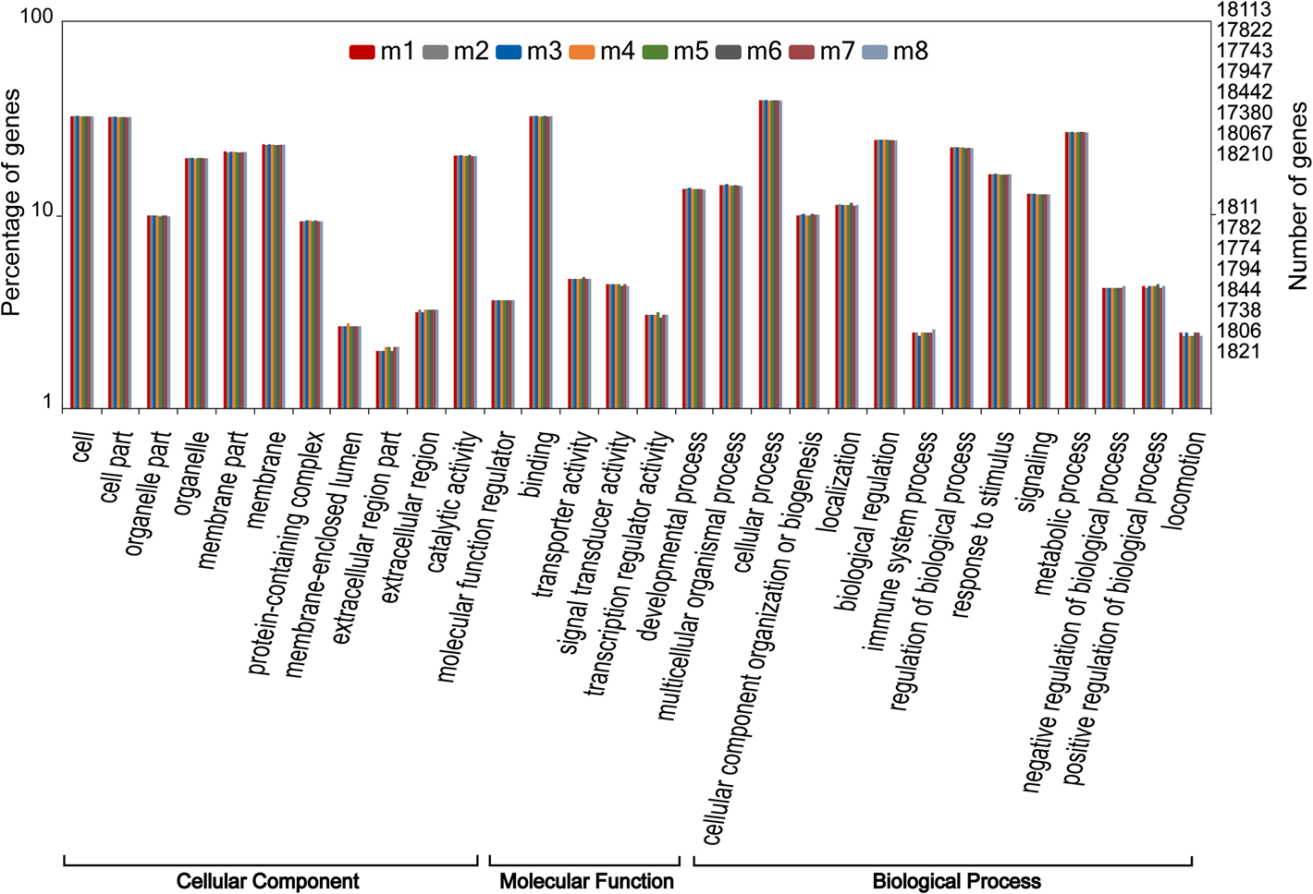
WEGO clustering of genes with SNPs

**Fig. 16:**
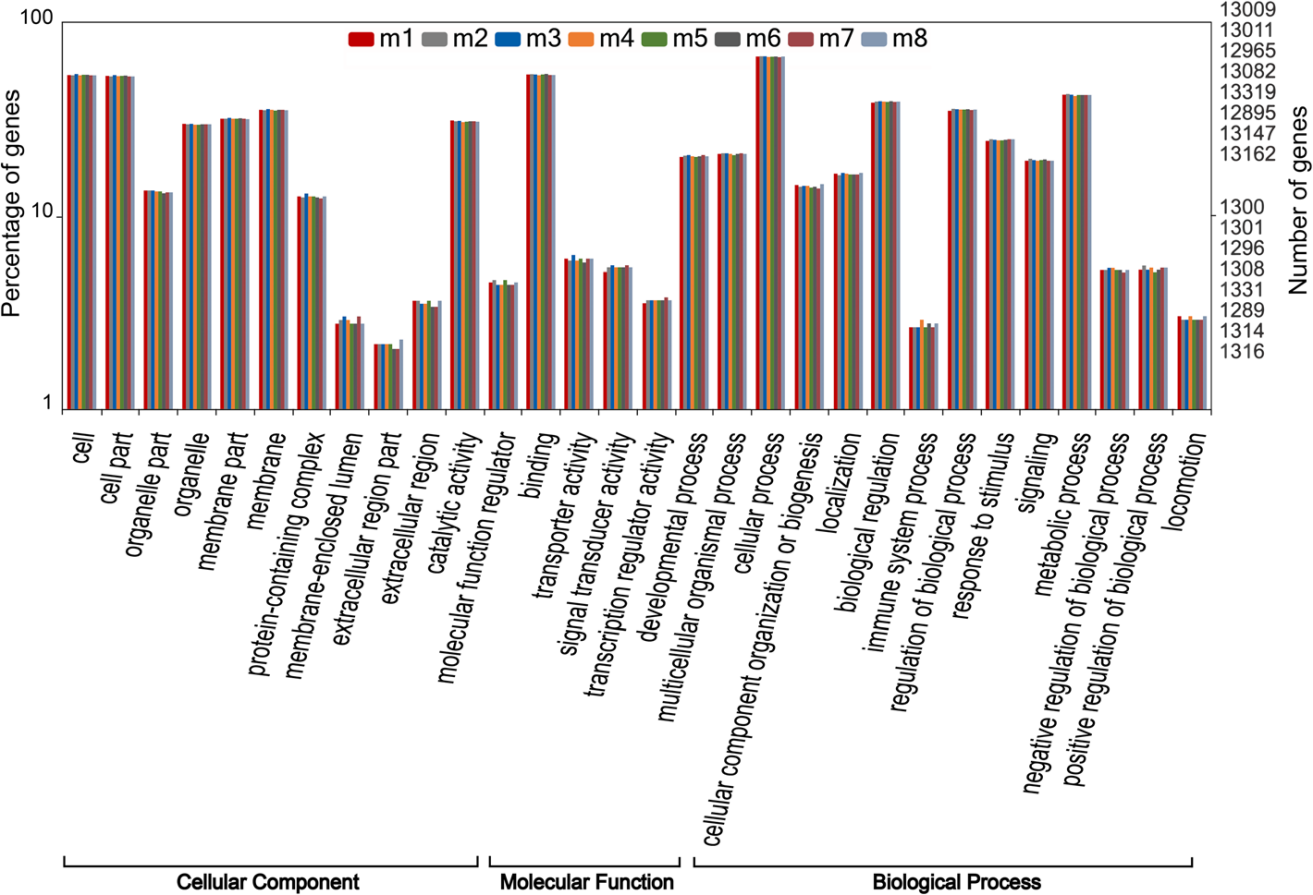
WEGO clustering of genes with InDels

In total, SNPs and InDels were located in 17,394~18,457 and 12,907~13,333 genes, respectively (Fig. 14). GO annotation was performed and plotted by WEGO for gene function clustering (Figs. 15 and 16). The results indicated that SNPs and InDels were distributed among different gene ontologies. In the cellular component ontology, the GO terms cell and cell part contained the majority of the mutant genes, with 34.3% SNPs on average and 34.7% InDels. GO terms associated with the extracellular region, such as extracellular matrix and extracellular space, had relatively lower numbers of the mutant genes. In the molecular function ontology, binding and catalytic activity contained higher mutation rates. Mutations in the binding term were composed of 34.4% SNPs and 35.2% InDels, while the catalytic activity term was composed of 21.3% SNPs and 21.6% InDels. For the biological process ontology, cellular process, metabolic process and biological regulation had high gene mutation rates. Mutations in the cellular process term were composed of 41.6% SNPs and 42.3% InDels. Metabolic process contained 28.3% SNPs and 28.2%. Biological regulation included 25.7% SNPs and 26.5% InDels.

### Mutant genes pertaining to the growth and immune pathways

We selected five genes that were related to growth (*mstn*, *myod*) and immune regulation (*tp53*, *mhc1zaa, mhc2dab*) to investigate the distribution of SNPs. In the eight ARTP-treated individuals, 5 to 33 SNPs were then detected (Table 7). Five SNPs of *tp53* gene, and one SNP of *mstn* gene that detected in ARTP treated samples were selected for confirmation by using Sanger sequencing. The result indicated that these SNPs were only detected in ARTP treated samples, but not in controls, which was identical with result of whole genome sequencing (Table 8). Interestingly, a high percentage of SNPs were located in the exon and intron regions of *tp53*, *mhc1zaa* and *mhc2dab*. Such SNPs may be beneficial for the further molecular breeding, because it is possible that the SNPs will accelerate the selection of disease-resistant new varieties.

**Table 7:** Selected gene mutations in ARTP treated samples

Gene	Chromosome	Length (bp)	Group	Total No. of SNP	Exonic	Intronic	Downstream	Intergenic	Upstream
*mstn*	15	11,730	ARTP treated	11	1	/	1	8	1
			Control	3	/	/	1	2	1
*myod*	7	68,283	ARTP treated	19	/	6	5	4	4
			Control	3	/	1	/	2	
*tp53*	9	35,221	ARTP treated	33	5	28	/	/	/
			Control	14	7	7			
*mhc1zaa*	22	10,720	ARTP treated	13	/	9	/	4	/
			Control	2	/	/	/	1	1
*mhc2dab*	6	9736	ARTP treated	5	/	5	/	/	/
			Control	0	/	/	/	/	/

**Table 8:** SNP verified by Sanger sequencing

Gene	Locus	Primers 5′-3′	Size (bp)	SNP type	Detected in ARTP treated sample	Detected in control
*tp53*	9,685,724	F: TGAAGAGCATAGCCAGGAG	228	A > T	m8	/
		R: ACCCTGATTCTTTAGGCCT				
	9,704,973	F: TCAGGTATGGCTTCCTCAC	236	A > G	m5, m6, m8	/
		R: AAACAAGACTTACTCGGGAG				
	9,705,000	F: TCACTCAGGGGAACATGT	192	T > A	m5, m6, m7, m8	/
		R: TCAGGATGGTGAGGATCTG				
	9,705,895	F: TTCCTGTGAGTAATGATGCAGT	226	T > C	m5, m6, m7, m8	/
		R: ACATTTGGAATTGATTGCAAAGT				
	9,712,498	F: CCCATTTGTTCAGAACCGC	164	G > A	m5, m6, m7, m8	/
		R: TCCAGTGAGAACGATTGCC				
*mstn*	6,396,324	F: TCTCAGACCGTGATGTTTT	228	C > T	m2, m4, m7	/
		R: ATTCCTGGTGGTCTGTG				

## Discussion

ARTP is a novel, efficient, safe and environment-friendly mutagenesis tool with a higher mutation rate than those of traditional mutagens [17]. Although ARTP mutagenesis has been widely applied to microbial mutation breeding and proved its effectiveness, its application in aquaculture species has not been reported so far. In this study, we applied ARTP to mutation breeding of Japanese flounder for the first time by optimizing ARTP mutation conditions. The success and feature of the ARTP mutagenesis for the fish was confirmed by SNP and InDel calling at the genome level.

In microbial mutation breeding, lethality and positive mutation rate were used as indexes for determining the ARTP treatment conditions [31]. However, in our study, unlike the microbial mutagenesis, due to the difficulty of determining the positive fish mutation rate (because long period of the growth is required), we defined the abnormal rate as the index of ARTP treatment to the fish eggs and sperm.

Under the same ARTP power and working gas flow rate, ARTP treatment time is equivalent to the mutation dosage, and the optimal treatment time in this study was significantly longer than that for microbes. The reason might be the differences in sizes and structures between Japanese flounder eggs and microbes. The eggs of Japanese flounder are approximately 1 mm in diameter, but for most microbes, the diameter is measured in μm. For example, *Crypthecodinium cohnii* is 25 μm in long diameter [32], which is almost 40 times smaller than that of the Japanese flounder eggs. Thus, the relatively large size of the egg might require a higher ARTP plasma irradiation dosage, indicating that under a fixed input power of ARTP, a longer irradiation duration is required. For sperm, although the diameter of the head is approximately 1.5 μm, the high level of chromatin condensation might reduce the DNA-damage effect of ARTP treatment and give rise to the longer irradiation duration.

We also interestingly found that the nonshade treatment after 8 to12 min ARTP treatments of 40X diluted sperm could significantly increase the normal rate of hatched larvae. Effect of light on hatching process of sperm has little been known. The nonshade effect mentioned above might be explained by nucleotide excision repair (NER) or photoreactivation repair (PER) theory. These two mechanisms are two major repair pathways for UV-induced photo-lesions [33]. In fish, NER or PER have been studied in vivo [34] and in vitro [33]. In rainbow trout (*Oncorhynchus mykiss*) cell lines, PER repair much faster the UV lesions than NER [33]. NER or PER could also repair UV irradiated sperm [35]. Preventing UV irradiated sperm from visible light is crucial for induction success rate in artificial gynogenesis. However, ARTP has very low UV level, and whether NER or PER could be related to repair of the ARTP treated sperm is not clear, and needs further studies.

In this study, we used next-generation whole-genome sequencing technology to analyze the genetic mutations in ARTP-treated individuals. This study represents the first time that whole-genome sequencing technology has been used for ARTP-induced mutation of fish. Three female parents were from a homozygous clonal family with a genetic similarity of 1.00 and a homozygosity of 1.00 [27]. And a male doubled haploid with homozygosity of 1.00 was used as male parent [28]. The offspring obtained by normal fertilization of these parents were heterozygous clones, and the genetic similarity between the offspring was 1.00 [36]. We compared each ARTP treated sample m1-m8 with c1 first, then compared the results with c2, and then compared the results with c3. The final results were used for the next analysis. This not only removed the shared SNPs of m1~m8 to each control, but also removed the natural mutation occurred, because in nature, the mutation rate per generation was low, for mouse, SNP mutation rate was 4 × 10^− 9^, and 3 × 10^− 8^ for human [37]. For Japanese flounder, the estimate natural mutation of SNP per generation is about 546 [the genome size of Japanese flounder is 5.46 × 10^8^(546 Mb) [29], and mutation rate is set as 1 × 10^− 6^]. Such a low number of natural mutation could not affect the calculation of ARTP mutagenesis rate.

We found that the mutation rate in terms of SNPs and InDels was 0.064% on average at the genome level. Gene mutation rate is one of the main points of interest in studies of artificial mutation induction. In ENU-induced mutation of zebrafish [38] and γ-radiation/ENU-induced mutation of medaka [10, 39], the mutation rate was calculated using albino mutants, which carry mutations in the tyrosinase gene and are readily visible due to their red eyes. However, albino mutations are found in only a few species, not in all fish species in nature [1]. With the development of molecular technology, the mutation rate can be calculated as the frequency of mutated base pairs to the total length of specific genes using technology such as direct resequencing or high-resolution melting analysis [1, 4–6]. We used whole-genome sequencing with an average depth of 9.77X, and such a large scale increases the possibility for discovering more mutation loci. Although ARTP radiation is a physical process, the genes in the genome seemed to have an even opportunity to be mutated. However, we found in our study that different genes had different mutation rates after ARTP radiation. So, calculation of mutation rate based on a single gene or several genes could increase large errors, and the whole-genome scale of measure is a more reliable way to detect the effects of chemical or physical mutagenesis on gene mutation rate.

Among the mutations, SNPs were in the majority, indicating that point mutations were the main type of variation induced by ARTP treatment. Similar results were also found in rice (*Oryza sativa* L. ssp. *indica*) mutations induced by γ-radiation [40] and in ENU-induced mutations in medaka [4], fugu [1], and zebrafish [5]. In γ and X ray irradiation, other types of mutations were also observed. X ray irradiation could cause mutations such as single-nucleotide variants, InDels as well as copy number variants (CNVs) in mice [41]. CNVs and presence/absence variations were also detected in the genome of γ irradiated rice [40]. And in medaka, deletion of genome sequence could be induced by γ-radiation [10]. However, whether mutations like CNV etc. occurred in ARTP treated individuals are not clear, and need further study. Furthermore, WEGO clustering of mutant genes showed equal distribution of different gene ontologies between SNPs and InDels, as well as among the individuals. This result indicated that ARTP-treated individuals share similar survival and distribution patterns in terms of gene ontology.

## Conclusions

In conclusion, the ARTP mutagenesis method was established for breeding of Japanese flounder by optimizing the ARTP treatment time period and the genome mutation analysis of the mutated fish using whole-genome sequencing. This is the first report that the novel ARTP mutagenesis is an applicable method for breeding of fish species. This approach may play a significant role in commercial breeding for selection of economically important traits that would benefit the aquaculture industry.

## Methods

### Fish and gamete collection

The experiment was performed at Beidaihe Central Experimental Station, Chinese Academy of Fishery Sciences. Mature female and male Japanese flounders were reared in the conditions used by Hou et al. (2016) [28]. A second-generation homozygous clone family 3165 [27] was used for egg collection, and eggs were manually stripped and collected with 1000-ml glass beakers. In total, three clonal females were used for egg collection. Sperm was collected with a 5-ml syringe from one doubled haploid male by gentle pressure on the abdomen. The collected eggs and sperm were stored in darkness before use.

### Mutation by ARTP

For mutation of the fertilized eggs, the sperm was diluted with Ringer’s solution at a ratio of 1:40 *v*/v, added to eggs, mixed well and activated using 17 °C filtered seawater. The fertilized eggs were incubated at 17 °C until 60 min after fertilization and then treated with a pure helium-based ARTP mutation machine (ARTP-A, TMAXTREE Biotechnology Co., Ltd., Luoyang, China). Approximately 1800 eggs at metaphase of first mitosis were placed on a glass Petri dish with 1 mL sea water. The dish was then exposed to the plasma, and the ARTP mutation system was operated with the following parameters: radio-frequency (RF) power input of 120 W, helium gas flow rate of 10 L/min, treatment distance of 2 mm, and treatment time period of 1.5, 3, 6, 9, 12, 14, 25 and 30 min. After the ARTP mutation treatment, eggs were transferred to a 17 °C water bath until hatching.

For mutation using the sperm, 5 ml of Ringer’s solution-diluted sperm was placed on a glass Petri dish and treated by ARTP with operating parameters as follows: RF power input of 200 W, helium gas flow rate of 10 L/min, treatment distance of 2 mm, and treatment time period of 2, 4, 6, 8, 10, and 12 min for 1:40 diluted sperm (40X) and 4 and 10 min for 1:6 diluted sperm (6X). After ARTP treatment, the sperm was shaded or unshaded from visible light and used to fertilize eggs within 5 min. The fertilized eggs were transferred to a 17 °C water bath until hatching.

### Fertilization, hatch and abnormal rates

The fertilization, hatch and abnormal rates were calculated according to Hou et al. (2016) [28]. For mutation using sperm, the relative abnormal rate was calculated as the ratio of the abnormal rate in the ARTP treatment group to that in the control group.

### Fluorescence staining of ARTP-treated sperm

Both the ARTP-treated and control sperm were double-stained with 10 μg/ml Rh123 and PI for 10 min in darkness. For each group, 200 μl of sperm was stained. After staining, 20 μl of solution was placed on a slide and observed under a fluorescence microscope (Leica DM 4000B) with an excitation wavelength of 488 nm. At least 3000 stained spermatozoa were observed for each group.

### Morphological characteristics of ARTP-treated individuals

The ARTP-treated group (fertilized eggs with an ARTP treatment time of 25 min) was cultured under the same conditions as the control group. From day 0 to day 60, the larvae were reared in a 3-m^3^ aquarium for each group with flow-through seawater. At day 61, the fish of each group were transferred to a 25-m^3^tank. At day 90, 180 individuals each from the ARTP and control groups were randomly selected and measured for body weight, total length, head length, body height and caudal peduncle length.

### Whole-genome sequencing

At day 120 after hatching, eight individuals from the ARTP-treated group and three individuals from the control group were randomly selected, respectively, and the fin samples were cut. Genomic DNA was isolated from each fin sample using phenol-chloroform extraction [42]. Following quality assessment, the isolated genomic DNA was randomly fragmented into 350 bp by sonication. The fragmented DNA was then prepared for library construction using the TruSeq DNA Library Prep Kit HT (FC-121-2003, Illumina), and sequenced by Illumina NovaSeq 6000.

### SNP and InDel detection

Paired-end sequencing reads were mapped to the Japanese flounder reference genome [29] with BWA [43] using default settings. SAMtools [44] software was used to filter the unmapped and nonunique reads (parameter: rmdup), and the duplicated reads were filtered with the PICARD package (http://broadinstitute.github.io/picard). The raw SNP/InDel sets were first called by SAMtools with parameters as ‘mpileup -m 2 -F 0.002 -d 1000’, and then, these called sets were further filtered using the following criteria: mapping quality > 20, and the depth of the variate position > 4. After calling, the SNPs/InDels were annotated to the Japanese flounder reference genome [29] using the package ANNOVAR [45]. We compared each ARTP treated sample m1-m8 with C1 first, then compared the results with c2, and then compared the results with c3. The final results were used for the next analysis. This removed the shared SNPs or InDels of m1~m8 to each control. Gene Ontology (GO) clustering analysis of genes that contained SNPs or InDels was performed by WEGO 2.0 [46].

### PCR and sanger sequencing

To verify the SNPs that detected by whole-genome sequencing, five SNPs of *tp53* gene, and one SNP of *mstn* gene were selected. DNA from eight individuals of the ARTP-treated group and three individuals of the control group that used for whole-genome sequencing were used here for PCR amplification. The PCR cocktail was 15 μL in volume, containing 1.5 μL of 10 × buffer, 1.5 μL of Mg^2+^ (25 mmol/μL), 0.25 μL of dNTPs (10 mmol/μL), 0.15 μL of each primer (10 pmol/μL), 0.2 μL of *Taq* DNA polymerase (5 u/μL), 1 μL of DNA (30~50 ng), and 9.5 μL of ddH_2_O. PCR thermal cycles comprised of one cycle of pre-denature (95 °C for 3 min), followed by 35 cycles of amplification (94 °C for 15 s, 55 °C for 15 s, 72 °C for 30s), and a final extension step (72 °C for 3 min). Cloning of PCR products and Sanger sequencing were performed according to Jiang et al. (2011) [6].

### Statistical analysis

All ARTP treatment experiments were performed in triplicate, and the data are given in the format mean ± SD (standard deviation). The coefficient of variation (CV) was calculated as the ratio of SD to mean. The data for the optimization of ARTP treatment duration were analyzed by a one-way analysis of variance (ANOVA) followed by LSD multiple comparisons (*P* < 0.05). Paired t-tests were performed to compare the morphological characteristics between the ARTP-treated group and control group, as well as to compare different dilutions or shaded/nonshaded treatments in the sperm. For all statistical analyses, R software was used [47].

## Abbreviations

ARTP: Atmosphere and room temperature plasma
AVOVA: One-way analysis of variance
CNVs: Copy number variants
CV: Coefficient of variation
ENU: *N*-ethyl-*N*-nitrosourea
GO: Gene ontology
InDel: Insertion-deletion
LSD: Least-significant difference
NER: Nucleotide excision repair
PER: Photoreactivation repair
RF APGD: Radio-frequency atmospheric-pressure glow discharge
RF: Radio-frequency
SD: Standard deviation
SNP: Single nucleotide polymorphism
UV: Ultraviolet
